# Review

**Published:** 1862-10

**Authors:** 


### REVIEW.

*The half yearly abstract of the Medical Sciences; Edited by* W. H. RANKING, M. D, *Physician to the Norfolk and Norwich Hospitals, and* C. B. RADCLIFF, M. D., *Licenciate of the Royal College of Physicians of London. No.* 35, *January to July,* 1861. *Philadalphia:* LINDSAY & BLAKESTON.

Part I. Practical Medicine, Patholoy, and Therapeutics.

Part II. Surgery.

Part III. Midwifery and diseases of women and children; Reports on the Progress of the Medical sciences.

Rankin's half-yearly abstract of the Medical Sciences is the compendium of what has been written or discovered in the science or practice of medicine during the six months preceding its publication. This compilation embraces the principal heads, or general principles, of almost all new and valuable suggestions in medicine, surgery, and the collateral sciences; selections being made ftom the current medical literature of all nations. It is one of the most attractive publications for the physician, from which he may learn, at least in brief, the advances which are being made in his profession; he may almost "gain the rewards of diligence without suffering its fatigues."—Many valuable suggestions will be found in its pages, since it is prepared with especial reference to practical medicine, and contains papers interesting and valuable to every one engaged in the active duties of the profession.

